# Studying waiting time in pharmacy: A strategy for improving patient satisfaction

**DOI:** 10.1016/j.mex.2025.103282

**Published:** 2025-03-25

**Authors:** Rika Yulia, Ruddy Hartono, Mufida Indrayanti, Nur Palestin Ayumuyas, Fauna Herawati

**Affiliations:** a Department of Clinical and Community Pharmacy, Faculty of Pharmacy, University of Surabaya, Jalan Raya Kalirungkut, Surabaya, 60293, Indonesia; b Department of Pharmacy, Rumah Sakit Bhayangkara H.S. Samsoeri Mertojoso, Jalan Ahmad Yani No. 116, Surabaya, 60231, Indonesia; c Department of Pharmacy, Rumah Sakit Husada Utama, Jalan Prof. Dr. Moestopo No. 31-35 (Jl. Petojo), Surabaya 60131, Indonesia; d Department of Pharmacy, Rumah Sakit Umum Daerah Haji Provinsi Jawa Timur, Jalan Manyar Kertoadi, Surabaya, 60116, Indonesia

**Keywords:** Waiting time, Pharmacy, Pharmacies, Drug dispensing, A direct observation as a part of Lean Six Sigma with Time-Motion Analysis

## Abstract

This study analyzes waiting times for compounded and non-compounded medication services and identifies factors influencing prescription delays in public and private hospitals in Surabaya. Using a quantitative cross-sectional design, the study collects data in two phases: the first focuses on outpatients and their prescription data sheets, while the second involves pharmacists and pharmacy technicians through a questionnaire. The average waiting time for compounded prescriptions was 80 min in public hospitals and 36 min in private hospital. For non-compounded prescriptions, the averages were 64 min in public hospitals and 43 min in private hospital, with some cases as low as 28 min. These results exceed the Ministry of Health standards of 60 min for compounded drugs and 30 min for non-compounded drugs. The main factors influencing waiting times were the high workload of pharmacy staff during peak hours, the number of items in prescriptions, and insufficient pharmacy staff.

Patient satisfaction in the outpatient division is associated with

• Information technology,
• Standard operating procedures, and
• Human resources.

## Graphical abstract

**Figure:**
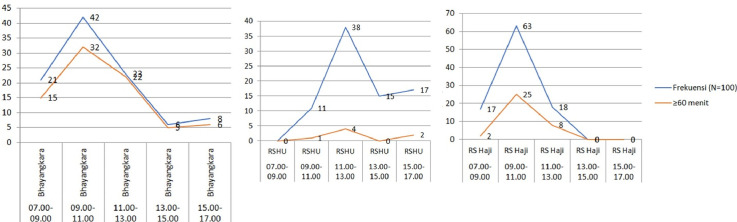

Specifications table

**Table:** 

Subject area:	Pharmacology, Toxicology and Pharmaceutical Science
More specific subject area:	Pharmacy Practice
Name of your method:	A direct observation as a part of Lean Six Sigma with Time-Motion Analysis
Name and reference of original method:	
Resource availability:	Data is available in https://repository.ubaya.ac.id/.

## Background

Long waiting times in pharmacies can lead to patient frustration and dissatisfaction [1]. Identifying bottlenecks and inefficiencies in the workflow can help streamline operations [2,3], making better use of resources [4,5] and improving service delivery. Efficient operations can reduce labor costs and other operational expenses [5], making the pharmacy more cost-effective. Pharmacies with shorter wait times are likely to have a better reputation [6,7], attracting more customers and improving business. Reducing waiting times can significantly enhance patient satisfaction and operational efficiency. Analyzing peak hours and patient demand helps allocate staff more effectively [1], ensuring adequate coverage during busy periods. Consistent processes for tasks like drug stock checking and service counter operations can reduce delays and improve efficiency [8]. Reducing paperwork and adopting electronic health records (EHRs) can speed up administrative tasks, allowing for quicker patient service [9]. Automated dispensing systems can take over time-consuming tasks like counting, pouring, and labeling prescriptions, freeing up staff to assist patients more quickly [10].

However, several factors should be considered when implementing strategies to reduce waiting time. Implementing EHRs and other digital tools raises concerns about data security and patient privacy, necessitating robust security measures. Integrating new technologies with existing systems can be complex, leading to compatibility issues, delays, and additional costs. Upgrading technology, hiring additional staff, and redesigning workflows can be expensive [1], which may be challenging for smaller pharmacies. Staff resistance to adopting new technologies or altering established workflows can slow down the implementation process and reduce the effectiveness of new strategies. Extensive training is often required for new systems and processes, which can be time-consuming and costly.

Patient satisfaction is closely associated with waiting times. National regulations set a minimum service standard for waiting times: 30 min for dispensing prescription medicine and 60 min for extemporaneously prepared medicinal products (compounded medication in prescription) [[11], [12]]. Studies have shown that reducing waiting times can significantly increase patient satisfaction [11]. However, some pharmacies still struggle to meet these standards [7]. The average duration of service during the prescription phase, especially for compounded medications, often results in longer preparation times compared to ready-made alternatives. Factors contributing to increased waiting times include high prescription volumes, insufficient human resources [13], medication procurement challenges, and limitations in compounding equipment [[14], [15], [16], [17]].

Six Sigma improves healthcare by reducing costs, medical errors, and wait times. Combines Lean's waste reduction with Six Sigma's process improvement to eliminate waste and reduce variability [18]. For improving existing processes, this study using DMAIC (Define, Measure, Analyze, Improve, Control) methodology begins with i. identifying problems and goals, ii. gathering baseline data (for example, from control charts), iii. finding root causes (assessing outcomes and effectiveness), iv. implementing solutions, and v. maintaining improvements.

Lean methodology optimizes the flow of value through processes by eliminating waste and emphasizing employee engagement and continuous improvement. It identifies types of waste like excessive work in progress, inefficient processes, and unused skills. Lean's culture includes ongoing training, encouraging participation, and maintaining standards for adaptability in today's fast-paced environment. The Six Sigma, on the other hand, focuses on improving quality by reducing defects using a structured, data-driven approach. Combining Lean and Six Sigma methodologies creates a robust framework for operational excellence by aligning process improvements with patient needs. This integrated approach streamlines operations, fosters a culture of continuous improvement, and transforms hospital pharmacy services to be effective, reliable, and patient-centric. Studies show that implementing Lean Six Sigma increases satisfaction rates, reduces medication expenditure, improves clinical pharmacy activities, and enhances the quality of pharmacy services by reducing errors, wait times, and optimizing resource management [19].

Queuing theory is the mathematical study of waiting lines or queues. It examines how lines form, how they function, and how they can be optimized. There is some process in queuing theory, i.e., arrival, dispensing (preparing product), giving/delivering product (including patient counseling time). Some variables in queuing theory are queue capacity, average waiting time, and average queue length [20]. Therefore, this study aims to evaluate waiting times for prescription services as a strategy to enhance the quality of pharmaceutical care within hospitals, ultimately improving the overall quality of hospital services.

## Method details

This study utilizes a descriptive cross-sectional design, with a focus on descriptive analysis. It is divided into two parts: the first part involves prescription outpatient patient's data serving as the primary material. The second part comprises healthcare professionals, specifically pharmacists and pharmacy technicians, who are surveyed using questionnaires for data collection.

Inclusion criteria for Phase I are as follows: 1. Compounded and non-compounded medication prescriptions for outpatient patients at public and private hospitals in Surabaya; 2. Prescriptions that are actively awaited by patients or their families on the day of data collection. Exclusion criteria include: 1. patients receiving medications via delivery; 2. patients with prescriptions for which not all medications were collected at the hospital; 3. patients who did not retrieve their medications on the specified day; and 4. patients who left their prescriptions unclaimed. The data collection method for the first part of the study commenced with patients receiving a barcode issued by their physician, which they subsequently presented to the pharmacy staff. Upon receipt of the barcode, the pharmacy staff initiated the process of measuring waiting times for prescription services. This timing began with the reconciliation of the prescription and continued until the patient received their completed medication.

Inclusion criteria for Phase II are as follows: pharmacists and pharmacy technicians at public and private hospitals in Surabaya who were active during the period of questionnaire distribution. Respondents who agreed to participate were required to sign an informed consent form.

The questionnaire sheet is divided into two sections: 1. respondent demographic data (name, age, occupation, and length of employment), 2. factors influencing prescription waiting times. The questionnaire was designed to gather the personal opinions of each pharmacist and pharmacy technician .

### Questionnaire development stage

To ascertain the validity and reliability of the questionnaire, validity and reliability tests were conducted. The validity assessment was based on the 2-tailed significance value, while reliability was measured using the Cronbach's Alpha method. A questionnaire is considered reliable if it achieves a Cronbach's Alpha value greater than 0.70 [21]. Additionally, the questionnaire is deemed valid if the 2-tailed significance value is <0.05.

In the analysis of the second part of the study, factors influencing prescription waiting times were evaluated using a modified Likert scale format. Responses were quantified through percentage calculations, allowing participants to respond across a range of levels (1–5). Each response was assigned a weight according to the following criteria to identify the most influential factors in the research: a. Score 5 for “Strongly Agree”, b. Score 4 for “Agree”, c. Score 3 for “Neutral”, d. Score 2 for “Disagree”, e. Score 1 for “Strongly Disagree”.

## Method validation

As part of the Lean Six Sigma initiative, the Time-Motion analysis validated the process improvements. By observing and recording the time for each workflow step, we identified inefficiencies and potential improvements. This analysis pinpointed bottlenecks and quantified how each step impacts overall efficiency. The study's results will help us reduce idle time and enhance productivity and quality Figs. 1a, b and 2.

**Fig. 1a:**
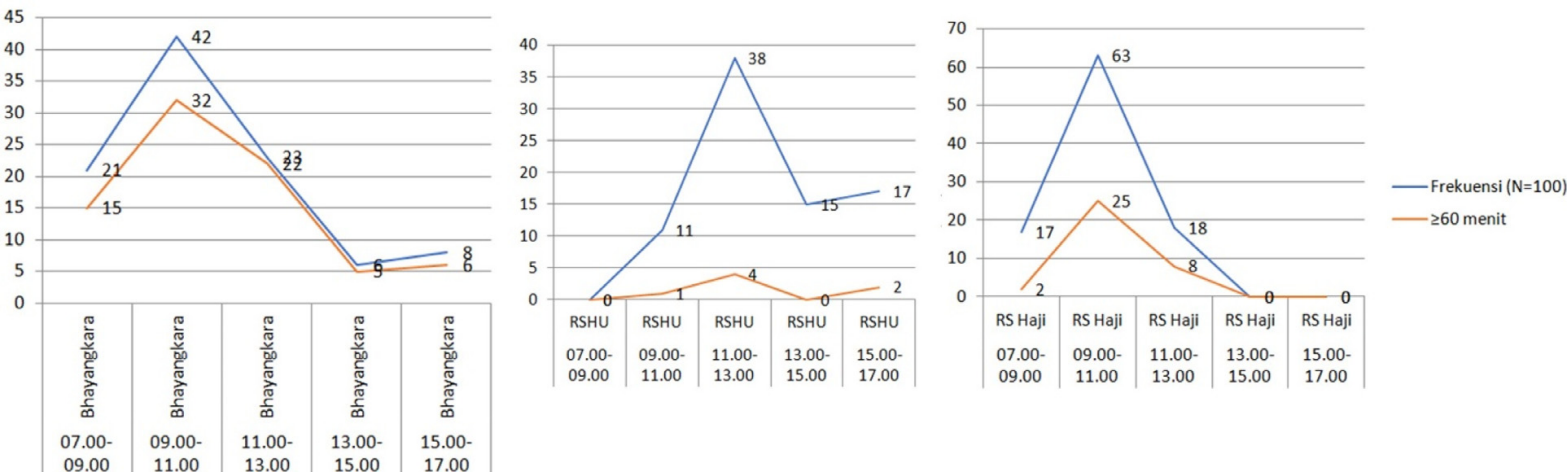
Impact of Queue Capacity on Compounded Prescription Processing.

**Fig. 1b:**
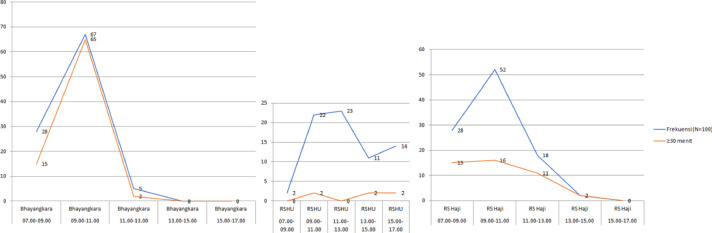
Impact of Queue Capacity on Non-Compounded Prescription Processing.

**Fig. 2:**
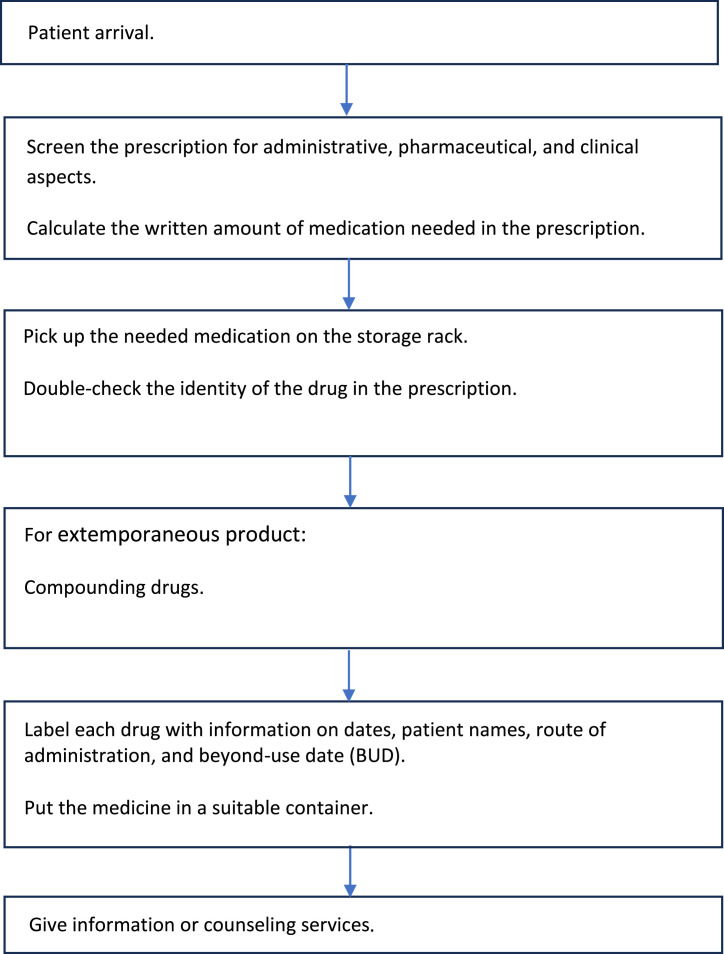
Flowchart of the Prescription Service Process.

## Discussion

Pharmacy drug preparation is vital and unique, requiring pharmacists to verify prescriber and patient data, ensure correct medication and dosage (especially for children), screen for interactions (notably with compounded drugs), and evaluate the benefits and safety for each patient (Fig. 2). The process must also be efficient and meet government standards to satisfy patient expectations and improve satisfaction Table 1, Table 2–3.

**Table 1:** Distribution of Prescription Profiles in Outpatient Department.

Outpatient Clinic	Number of Compounded Medication Prescriptions (*N* = 300)			Number of Non-Compounded Medication Prescriptions (*N* = 300)		
	Bhayangkara Hospital	Husada Utama Hospital	Haji Hospital	Bhayangkara Hospital	Husada Utama Hospital	Haji Hospital
Pediatric	9	50	12	2	34	4
Pulmonology	42	9	15	4	0	4
Internal Medicine	8	5	4	35	12	7
Psychiatric	27	3	21	-	0	4
Cardiology	2	3	1	17	2	12
Obstetrics	-	-	-	4	16	1
Neurology	6	4	14	14	2	7
Gastro-hepatology	-	-	7	-	-	12
ENT	6	11	10	-	3	7
Surgery	-	-	3	1	6	10
Other*	-	15	13	23	25	32

⁎ Number <10: general practice, urology, endocrine, dermatology & venereology, dental, ophthalmology, orthopedic, physical medicine and rehabilitation, orthopedi, growth and development, oncology gynecology.

**Table 2:** Frequency Distribution of Prescriptions by Service Hour.

Service Hour	Hospital	Number of Compounded Prescriptions			Number of Non-Compounded Prescriptions		
		Frequency (*N* = 100)	≥60 min	<60 min	Frequency (*N* = 100)	≥30 min	<30 min
07.00–09.00–	Bhayangkara Hospital	21	15	6	28	15	13
	Husada Utama Hospital	0	0	0	2	0	2
	Haji Hospital	17	2	15	28	15	13
09.00–11.00	Bhayangkara Hospital	42	32	10	67	65	2
	Husada Utama Hospital	11	1	10	22	2	20
	Haji Hospital	63	25	38	52	16	36
11.00–13.00	Bhayangkara Hospital	23	22	1	5	2	3
	Husada Utama Hospital	38	4	34	23	0	23
	Haji Hospital	18	8	10	18	11	7
13.00–15.00	Bhayangkara Hospital	6	5	1	-	-	-
	Husada Utama Hospital	15	0	15	11	2	9
	Haji Hospital	-	-	-	2	2	0
15.00–17.00	Bhayangkara Hospital	8	6	2	-	-	-
	Husada Utama Hospital	17	2	15	14	2	12
	Haji Hospital	-	-	-	-	-	-

**Table 3:** Questionnaire Assessment Results on Waiting Time Factors.

No	Question	Likert Scale Score		
		Bhayangkara Hospital (*N* = 32)	Husada Utama Hospital (*N* = 31)	Haji Hospital (*N* = 13)
	Human Resource Factors			
1	Adequate staffing levels in the outpatient installation	4.00	3.65	2.92
4	Tendency of pharmacy staff to delay the dispensation of medications to patients	2.56	1.90	2.00
5	High workload of pharmacy staff in the outpatient installation	4.19	3.65	3.61
9	Lack of skills among pharmacy staff in the outpatient installation when processing prescriptions	2.13	2.13	2.69
15	Difficulty in contacting prescribers for confirming medications on prescriptions	3.28	2.00	3.00
	Equipment Factors (availability of medications included)			
2	Inconsistent availability of prescribed medications in the outpatient installation	3.75	2.94	2.61
3	Comprehensive availability of equipment for compounding medications	3.34	3.87	3.77
6	Challenges with illegible handwriting on prescriptions during transcription	2.81	2.39	2.61
7	The impact of high item volume in prescriptions on processing times	4.06	4.06	4.07
12	Surge in compounded prescription requests during peak periods	3.62	2.68	3.76
	Facility and Infrastructure Factors (technology, standard operating procedures (SOPs), and facilities included)			
8	Implementation of computerized systems in outpatient pharmacy installations enhances the efficiency of prescription processing	3.84	3.68	4.30
10	Constraints of space in outpatient pharmacy installations for effective prescription services	2.19	4.00	3.46
11	Lack of established standard operating procedures (SOPs) for prescription services	2.97	2.42	1.84
13	Prolonged duration of prescription screening processes conducted by pharmacy staff	1.91	2.90	2.84
14	Robust information management systems can significantly accelerate prescription processing in outpatient settings	3.47	2.90	3.77

Description:

Scale 1: Strongly Disagree.

Scale 2: Disagree.

Scale 3: Neutral.

Scale 4: Agree.

Scale 5: Strongly Agree.

Domain Description:

1. Human Resource Factors: question number 1, 4, 5, 9, 15.

2. Equipment Factors (availability of medications included): question number 2, 3, 6, 7, 12.

3. Facility and Infrastructure Factors (technology, standard operating procedures (SOPs), and facilities included): question number 8, 10, 11, 13, 14.

A study of the lean approach identified value-added (VA), non-value added (NVA), as well as the waste time (WT) and reported that the time needed by the patient to get the drug was 36 min; 13, 17, and 6 min, respectively [22]. In this study, value-added (VA) activities are only producing products (including labeling) and services (drug information).

Extemporaneous compounding is performed when a combination of medications or doses specific to the individual patient is required [[12], [23]]. Many prescriptions are compounded in large quantities in the pulmonary unit because patients with chronic respiratory diseases require many medications, for example, beta-receptor antagonists, corticosteroids, anticholinergics, phosphodiesterase-4 (PDE) inhibitors, and mucolytics [24]; in pediatric patients, a compound prescription is needed because a dose is required that is appropriate for the child's age and weight.

The reason for the accumulation of prescriptions at these hours is the number of outpatient clinics that are open simultaneously, resulting in the accumulation of prescriptions during peak hours, 09.01–11.00 in private hospital and 11.00–13.00 in public hospitals. Therefore, additional pharmacy staff is needed during these hours to reduce the prescription bottle neck and shorten the waiting time for services. A study in Indonesia stated that the most influential factor in the waiting time for prescription services is human resources, which includes a shortage of pharmacy staff and a lack of capacity in prescription screening [25]. Delays in the drug delivery process will lead to an accumulation of patients in the waiting room, creating an inhospitable environment and may affect the efficiency of the service. Therefore, a fast response time for outpatient services is needed to improve service quality.

The large number of prescriptions received each day encourages the pharmacist and pharmacy technicians to process them immediately. The outpatient pharmacy department in public hospitals receives an average of 468 prescriptions per day, both compounded and non-compounded. To improve the quality of hospitals, outpatient services must be fast but accurate [26]. The number of different drug items in each prescription is the second factor that causes long waiting times for prescription services at the pharmacy department in the hospital. There are 1 to 8 items in compounded and non-compounded drug prescriptions. The higher the number of drug items in the prescription, the longer the waiting time for prescription services. The average number of drug items per best prescription sheet according to WHO estimates (1993) is 1.8–2.2 items [27]. This study showed that the average number of items per prescription form was 2–3, which exceeded the WHO best estimate. This value indicates a rather high tendency to polypharmacy due to the large number of prescriptions with >2 drug items. In addition, due to chronic diseases, the pharmacy has to dispense 30 to 60 capsules for the patient to use for one month (30 days).

Facilities and infrastructure factors, including technology, standard operating procedures (SOPs), and dispensing rooms, are highly influential in improving the efficiency of prescription services. Computerized systems have a significant positive impact by reducing human error, speeding up the process of transcribing prescriptions, monitoring inventory, and delivering medications to patients. This technology support also optimizes the performance of pharmacy staff, allowing them to focus on more complex tasks. The public and private hospitals in this study have implemented e-prescriptions to ensure that the prescription transcription process runs smoothly and efficiently. Good prescription legibility is essential to prevent errors in medication administration. The effective use of information technology speeds up prescription services, allows pharmacy staff to access information quickly, processes prescriptions more efficiently, and reduces manual errors.

In conclusion, various strategies can be implemented to address the length of the service process provided to patients, such as increasing human resources, analyzing the waiting time per patient, analyzing the completeness of health facilities, and analyzing the level of community needs for the length of health services [28]. The number of pharmacy staff is one of the supporting factors for the providing of maximum pharmaceutical services [29], so that the waiting time for patients to receive medicines is not long and increases patient satisfaction [30,31].

### Limitations

There are multiple steps in pharmacy drug preparation, and the time recorded doesn't cover every detailed activity. Recording the time for each step encourages the development of strategies to improve time efficiency. Currently, time is recorded only when the pharmacy accepts the prescription and when the patient receives the medicine. For thorough analysis and to identify bottlenecks, time should be recorded at every step. However, self-assessment by the pharmacy department staff on the situation and obstacles provides a clearer picture for further analysis.

## CRediT author statement

**Fauna Herawati**: Conceptualization, Methodology, Software. **Ruddy Hartono**: Validity tests, data curation, Writing —Original draft preparation. **Mufida Indrayanti**: Visualization, Investigation. **Rika Yulia**: Supervision. **Nur Palestin Ayumuyas**: Software, Validation. **Fauna Herawati**: Writing- Reviewing and Editing.

## Ethics statements

The study was conducted in accordance with the Declaration of Helsinki and approved by the Institutional Review Board (or Ethics Committee) of Komite Etik Penelitian Kesehatan Rumah Sakit Bhayangkara H.S Samsoeri Mertojoso (protocol code 16/VI/2024/KEPK/RUMKIT, approval date: 28 June 2024); Rumah Sakit Husada Utama (protocol code 18/KEP-RSHU/VI/2024, approval date: 10 June 2024); and Rumah Sakit Daerah Haji Provinsi Jawa Timur (protocol code 445/107/KOM.ETIK/2024, approval date: 30 May 2024). Participant consent was obtained in writing along with the questionnaire, and the analysis used anonymous data.

## Declaration of competing interest

The authors declare that they have no known competing financial interests or personal relationships that could have appeared to influence the work reported in this paper.

## Data Availability

Data will be made available on request.
